# Management of multiple common bile duct stones via laparoscopic transcholedochal exploration: an observational study

**DOI:** 10.3389/fsurg.2026.1682591

**Published:** 2026-05-21

**Authors:** Raj Man Dongol, Ananya Singh Bogati, Durga Neupane, Lalijan Awale, Shailesh Adhikary, Narendra Pandit

**Affiliations:** 1 Department of Surgery, BP Koirala Institute of Health Sciences, Dharan, Nepal; 2 Department of General Surgery, Nepal Medical College, Kathmandu, Nepal; 3 Department of Surgical Gastroenterology, Birat Medical College, Biratnagar, Nepal

**Keywords:** Laparoscopic common bile duct exploration (LCBDE), common bile duct (CBD), transcholedochal, choledochoscope, gallstones

## Abstract

**Objective:**

The study is an attempt to assess the feasibility of laparoscopic common bile duct exploration (LCBDE) in patients with multiple common bile duct (CBD) stones.

**Methods:**

A prospective observational study was conducted over 1 year (2017–2018). Patients with gallbladder *in situ*, bile duct stones (>1), and a dilated duct (>7 mm) were included. CBS stones and duct size were confirmed by ultrasound, computed tomography, and/or magnetic resonance cholangiopancreatography. Laparoscopic cholecystectomy and bile duct exploration (transcholedochal) were performed using a four-port technique. Patients were evaluated through regular follow-up for 6 months and required information was collected. The primary outcome was stone clearance rate. The secondary outcomes were operative time, postoperative complications, open conversion, and length of hospital stay.

**Results:**

A total of 31 patients were enrolled. The mean age was 44.03 ± 13.91 years with a male-to-female ratio of 2.87:1. The mean number of CBD stones was 4.62 ± 1.91. LCBDE was technically successful in 27 patients (87.09%), while 4 (12.9%) required open conversion. The failure rate of on-table choledochoscopy for complete stone clearance was 6.4%. The mean operative time in the successful laparoscopic group was 110.96 ± 20.15 min. No intraoperative complications were encountered. A T-tube was placed in 23 patients (74.19%), while 7 (22.58%) underwent primary closure of the CBD. One patient required Roux-en-Y hepaticojejunostomy for concomitant hepatolithiasis. Five patients (16.1%) developed postoperative complications (4 minor and 1 major). The mean length of hospital stay was 4.09 ± 1.10 days. On follow-up, there was no evidence of retained/residual stone. There was no postoperative (30 and 90 days) mortality. The stone clearance rate was 100%.

**Conclusion:**

The study showed that transcholedochal LCBDE is a safe procedure in terms of stone clearance rates, with acceptable perioperative complications and conversion rates for multiple CBD stones.

## 1 Introduction

The incidence of choledocholithiasis ranges from 9% to 16% in patients with cholelithiasis (1). Its incidence increases to over 80% in people over 90 years of age (2). It is the second most frequent complication of common bile duct (CBD) disease (3). There are numerous ways of dealing with CBD stones: (a) wait and watch for spontaneous passage of small stones; (b) open CBD exploration; (c) endoscopic retrograde cholangiography with sphincterotomy and stone extraction; and (d) transcystic or transcholedochal laparoscopic common bile duct exploration (LCBDE). Open CBD exploration was the procedure of choice during the era of open cholecystectomy. With the advancement of minimally invasive surgery, endoscopic retrograde cholangiopancreatography (ERCP) with or without sphincterotomy, performed preoperatively or postoperatively with laparoscopic cholecystectomy, emerged as the preferred treatment. However, ERCP combined with laparoscopic cholecystectomy is a two-stage procedure associated with risks of complications, including pancreatitis, bleeding, duodenal perforation, and prolonged hospital stay (4). The introduction of LCBDE has made it possible to avoid the drawbacks of both a two-stage procedure (preoperative ERCP + laparoscopic cholecystectomy) and open CBD exploration, with a benefit of a shorter hospital stay (1).

 Laparoscopic CBD exploration is now considered the most cost-effective and accessible option compared with other approaches. Several studies have demonstrated that LCBDE has numerous advantages over the other approaches in terms of clinical and economical outcomes (5). LCBDE has emerged as a minimally invasive, cost-effective technique for managing CBD stones. The present study aimed to assess the effectiveness of LCBDE in patients with multiple CBD stones at our center, focusing on stone clearance rate, operative time, open conversion rate, and perioperative outcomes. This study has been reported in line with the STROBE guidelines.

## 2 Materials and methods

This was a prospective observational study conducted over 1 year (2017–2018) at the Department of Surgery at a tertiary care hospital of Nepal. The study was approved by the Institute Review Committee and informed consent was obtained from all patients. All patients diagnosed with multiple common bile duct stones (more than 2 CBD stones) during this period were included in the study. Patients presenting with any of the following conditions were excluded from the study: acute cholangitis, acute pancreatitis, cirrhosis, liver mass, empyema gallbladder or perforation, pregnancy, recurrent choledocholithiasis, previous upper gastrointestinal surgery, or contraindications to general anesthesia.

Patient demographics and the presence of jaundice were recorded. Ultrasonography was the first-line imaging modality. Contrast-enhanced computed tomography (CT) scan of abdomen and magnetic resonance cholangiopancreatography (MRCP) were performed as required. Laparoscopic cholecystectomy was performed using the four-port technique. All laparoscopic CBD explorations were carried out through the transcholedochal route. Following spontaneous extrusion of stones through the choledochotomy incision, a choledochoscope was utilized to confirm the presence of preoperatively diagnosed stones, identify any unsuspected stones, and facilitate complete removal. The passage of pressurized saline through a side working port of the choledochoscope facilitated clearance of small stones and particulate matter and ensured all stones were removed. Other techniques included Fogarty balloon dilatation, Dormia basket, and grasping forceps. After removing all visible stones, final checking for complete stone clearance was performed by visualizing the proximal and distal ducts till the ampulla through the choledochoscope. On-table choledochoscopy is a reliable and accurate method for assessing CBD stones, as it allows direct visualization. In contrast, intraoperative cholangiography relies on imaging findings, which can sometimes be difficult to interpret for stone detection. Closure of the choledochotomy was performed either primarily or over the long limb of a T-tube (siliconized polyvinyl chloride Kehr's T-tube, 14 Fr) using interrupted 3–0 absorbable polyglactin sutures, based on intraoperative assessment and patient factors. We recoded operative findings, including size of the duct and stone, number of stones, and presence of difficult stones. A T-tube cholangiogram was performed on 10th postoperative day. T-tube removal was done after 4 weeks if no residual stones were detected. Patients were evaluated via regular follow-up after discharge at 1, 2, 4, and 6 weeks, and at 6 months. Follow-up assessments of patients included evaluation of history, laboratory findings, and imaging studies to detect any retained or residual CBD stones. History focused on clinical features suggestive of CBD stones, while laboratory evaluation emphasized liver function tests (LFTs), particularly alkaline phosphatase (ALP). Initial imaging was performed with abdominal ultrasonography. If patients presented with suspicious clinical history, deranged liver function tests—especially elevated ALP—or sonographic findings such as a dilated CBD, further imaging with CT abdomen or MRCP was advised for confirmation and stone assessment.

The primary outcome measure of this study was complete clearance of CBD stones using laparoscopic exploration. The secondary outcomes were open conversion, presence of residual stones at follow-up, and operative complications. All data were entered into Microsoft Excel and converted to SPSS version 20. Descriptive statistics were applied, including mean, median, frequency, and percentage, as appropriate.

## 3 Results

Out of 58 patients with choledocholithiasis, 36 patients were diagnosed with multiple CBD stones. Five patients were excluded from the study: two patients with past history of laparoscopic cholecystectomy, two with acute cholangitis, and one patient with pregnancy. Ultimately, 31 patients completed the treatment and follow-up. The mean age of the patients was 44 years, with a female predominance (74%). Obstructive jaundice due to CBD stones was seen in 15 patients (48%). The demographic and clinical profile of the study group is summarized in Table 1. Apart from ultrasound, contrast CT was performed in five (16%), and MRCP was done in 12 patients (39%) prior to surgery.

**Table 1:** Demographic and clinical profile of the study group.

Characteristics	Variables	Total patients (*n* = 31)	Percentage (%)
Age group (years)	<30	5	16.12
	30–60	15	64.51
	>60	6	19.35
Mean age ± SD (min–max)	44.03 ± 13.91 (18–70 years)		
Female to male ratio		2.87:1	
Body mass index (BMI)	<18.5	3	9.67
	18.5–24.9	22	70.96
	25–29.9	6	19.35
Mean BMI ± SD (min–max)	23 ± 2.3 (18–27.5 kg/m^2^)		
Comorbidities	Hypertension	4	12.9
	Obstructive airway disease	1	3.2
	Diabetes	1	3.2
Presenting complaints	Pain abdomen	29	93.54
	Jaundice	15	48.38
	Fever	4	12.9

Laparoscopic cholecystectomy with laparoscopic common bile duct exploration was technically successful in 27 patients (87.09%). Four patients (12.9%) required conversion to an open procedure due to frozen Calot's triangle with inability to achieve critical view of safety (1), impacted stone (1), associated left hepatolithiasis with choledochoduodenal fistula (1), and impacted left hepatolithiasis (1). In the case of left hepatolithiasis with choledochoduodenal fistula, we could not retrieve the stone which was impacted at the left duct; therefore, repair of choledochoduodenal fistula with omental plugging was performed along with T-tube placement. The patient underwent Roux-en-Y hepaticojejunostomy at a later date. In another case with left hepatolithiasis, as we could not retrieve the stone due to impaction, Roux-en-Y side-to-side hepaticojejunostomy was performed. As we could not retrieve stone in two patients, the failure rate of choledochoscopy was 6.45%.

The mean operative time was 117 ± 25.2 min (range 90–210 min). The mean operative time of “successful laparoscopic group” was 110.96 ± 20.15 min (range 90–150 min), whereas in the open group, it was 161.25 ± 35.42 min (range 125–210 min), which was significantly longer (*p* < 0.05). In our study, all patients had concomitant gallstones. No complications were encountered intraoperatively. The mean size of the CBD stones was 10.9 ± 4.2 (6–16 mm). The mean CBD diameter was 10.51 ± 4.4 (8–15 mm) and the mean number of stones was 4.62 ± 1.91 (3–10). The mean duration of postoperative hospital stay was 4.09 ± 1.10 (3–8 days). Detailed intraoperative findings are summarized in Table 2.

**Table 2:** Intraoperative findings.

Intraoperative findings	Categories	Patients (*n* = 31)	Percentage (%)
Size of CBD stone Mean ± SD (min–max)	6–10 mm	14	45.16
	>10 mm	15	48.38
	Impacted stones (unretrievable)	2	6.45
	10.9 ± 4.2 (6–16 mm)		
Location of stone in bile duct	Proximal	8	25.80
	Distal	20	64.51
	Both proximal and distal	3	9.67
CBD diameter Mean ± SD (min–max)	8–10 mm	15	48.39
	11–15 mm	14	45.16
	>15 mm	2	6.45
	10.51 ± 4.4 (6–15 mm)		
Mean operative time	110.96 ± 20.15 (90–150 min)		
Number of stones Mean ± SD (min–max)	3–6	27	87
	>6	2	6.45
	Impacted stones (unretrievable)	2	6.45
	4.62 ± 1.91 (3–10)		

A T-tube was placed in 23 patients (74.19%), while 7 patients (22.58%) underwent primary closure of the CBD. The choice between T-tube placement and primary closure was based on the presence of jaundice, load of CBD stones, intraoperative manipulation, and choledochoscopy findings. In patients with absence of jaundice, fewer than 5 CBD stones, minimal intraoperative CBD manipulation, adequate CBD diameter (at least 8 mm), and clear visualization of biliary tract with complete stone clearance on choledochoscopy, primary closure of CBD was performed. Of 23 patients who had undergone T-tube placement, cholangiogram revealed that 22 patients (95.65%) had normal findings, while there was a filling defect in one patient (3.22%) due to an impacted stone in the left hepatic duct, which we could not retrieve intraoperatively. T-tube removal was performed at 4 weeks in 18 patients (58.06%) and at 6 weeks in 5 patients (16.12%). The only major complication observed was bilioma following T-tube removal in one patient (4.34%), which was successfully managed with percutaneous catheter drainage.

Out of 31 patients, 5 (16.12%) developed postoperative complications (four minor and one major, i.e., bilioma), as shown in Table 3. At 6 months of follow-up, clinical evaluation, liver function tests, and abdominal ultrasonography did not reveal any relevant clinical findings, elevated ALP, or dilated CBD stones. Therefore, no evidence of retained or residual CBD stones was observed in any patient, and the stone clearance rate was 100%. The overall morbidity rate was 16%, with morbidity in successful laparoscopic group limited to 7.4%. There was no postoperative mortality at 30 and 90 days.

**Table 3:** Postoperative complications.

Morbidity (Clavien–Dindo class)	Group	*n* = 31	%
Atelectasis (Grade I)	Conversion to open	1	3.22
Port site infection (Grade II)	Laparoscopic	1	3.22
Paralytic ileus (Grade I)	Conversion to open	1	3.22
Bilioma (Grade IIIa)	Laparoscopic	1	3.22
Mild acute pancreatitis (Grade I)	Conversion to open	1	3.22

## 4 Discussion

Choledocholithiasis can lead to serious complications such as acute cholangitis and pancreatitis. It is thus critical to have improved and standardized processes for the diagnosis and treatment of CBD stones. Endoscopic treatment of stones is associated with high complication rates (19%), failure rates (10%–15%), and mortality rates (3%), depending on expertise and medical centers (6). Different studies have shown that LCBDE is associated with reduced hospital stay, lower economic burden, and less risk of morbidity (1, 3). In terms of diagnosis, transabdominal ultrasonography is the first-line, inexpensive, non-invasive investigation, with a sensitivity of 25%–63% and specificity of 95% (7). CT scanning offers a sensitivity of 87% and specificity of 97% (8). MRCP is a non-invasive and accurate imaging modality with 95% and 97% sensitivity and specificity, respectively (8). ERCP—long considered the gold standard for evaluating CBD stones—has a sensitivity of 90%–95% and specificity of 92%–98% (9). All LCBDE procedures in our study were performed by the transcholedochal route. The number, size, and location of CBD stones, as well as the diameter or anatomy of CBD and the cystic duct, help in deciding between transcystic and transcholedochal approaches. The transcholedochal approach is usually reserved for ducts of at least 8–10 mm in diameter, stones larger than the cystic duct, more than five bile duct stones, cystic ducts with low and medial junctions, and cases with hepatolithiasis (10). In a study by Chander et al., the transcholedochal route was considered to be a better option for Asian patients with multiple, large stones and dilated CBD (11). The transcholedochal route is also chosen based on surgeon preference.

In our study, the mean operative time in the successful laparoscopic group was similar to that seen in other studies (2). The mean operative time in the open conversion group in our study was significantly higher compared to the successful laparoscope group (*p* < 0.05). The operating time largely depends on anatomy, adhesions in and around the Calot's triangle or lesser omentum, facilities available, and the associated learning curve. The conversion rate of 12.9% in our study was slightly higher than rates reported in other studies, where it varied from 4% to 10% (3, 5, 12, 13). The reasons for conversion in these studies were similar to ours—the presence of dense adhesions/ fibrosis, intrahepatic stones, and large and impacted stones.

The mean duration of hospital stay in our study was 4.09 ± 1.1 days, which is comparable to results seen in other studies (range: 3–5.1 days) (5, 13). The mean hospital stay was also longer in various other studies, ranging from 6.4 to 15.6 days (3, 5). CBD closure over a T-tube was performed using interrupted sutures. A study conducted by Liu et al. showed that CBD closure using a running barbed suture technique was associated with short total operating time and hospital stay as compared to interrupted sutures (14).

One patient (4.34%) developed major complications (bilioma) following T-tube removal, which was managed successfully with pigtail catheter drainage of collection. No complications were observed in patients who underwent primary closure of the CBD. Several studies have shown that the duration of operation and length of hospital stay are shorter in the primary closure group compared with the T-tube group (3, 4). T-tube increases the risk of morbidity. Therefore, given the potential for T-tube–related complications, its use should be carefully individualized and judiciously considered for each patient. Zhang et al. suggested that postoperative T-tube drainage is unnecessary for decompression of biliary tree, which was associated with longer operative time, longer postoperative hospital stay, higher cost, and higher complication rates (T-tube group: 32.6%, primary closure group: 19.14%, *p*−0.138) (3). No intraoperative complications were observed, except for minor bleeding. Five patients (16.12%) developed postoperative complications. Among these complications, port-site superficial infection and bilioma were seen in the successful laparoscopic group, while atelectasis, paralytic ileus, and acute (mild) pancreatitis were seen in the conversion group. These complications were similar to those reported by other authors, with morbidity rates ranging from 2.66 to 16.17% (3, 5, 11, 12).

At 6 months of follow-up, there were no retained stone in any patients (stone clearance rate of 100%), comparable to other studies (5, 15). Some studies have reported that laparoscopic CBD exploration is associated with increased risk of retained stones compared with open exploration. Some studies have reported retained stones rates of 5%–10% in patients after laparoscopic procedures (16, 17). Apart from this, biliary peritonitis (2.15%), umbilical hematoma (1.51%), hemoperitoneum/cystic duct leakage (3.8%), and acute coronary syndrome (3.3%) are some of the complications seen in other studies, but not in ours (3, 13, 14, 18). Given the limited duration (6 months) of follow-up, we cannot comment on the incidence of late complications like recurrent stones and CBD strictures. Studies have reported late stone recurrence rates of 1.8%–2% (12, 16).

In a study by Topal et al., involving 113 consecutive patients, laparoscopic stone clearance of the bile duct was successful in 91.8% of cases, with a mean duration of hospital stay of only 2 days (19). There was no postoperative mortality (30 and 90 days) in our study. However, postoperative mortality, though minimal, was observed in studies conducted by Chander et al. (0.7%) and Paganini et al. (0.6%) (11, 16). Morbidity rates have also been shown to be lower in the LCBDE group (3.7%) than in the open surgery group (6.7%) (20).

Overall, laparoscopic CBD exploration is considered to be a safe and effective procedure and has better outcomes than endoscopic and open surgery in terms of hospital stay, postoperative pain, and cosmesis (21, 22). In cases with multiple CBD stones and CBD of 8 mm or more in diameter, the availability of intraoperative cholangiography and flexible choledochoscopy is advantageous (23). Some studies have also noted that formation of strictures is one of the drawbacks of LCBDE. It has also been seen that as the stone-to-cystic duct ratio increases—and size and number of CBD stones increase, and the diameter of the cystic duct decreases—there is increased likelihood of inadequate stone clearance (24). However, studies have suggested that if skilled manpower is available, all patients with CBD stones, excluding those with cholangitis, can be offered LCBDE. In fact, LCBDE with primary duct closure has been found to be significantly better that T-tube drainage in terms of operation time, total postoperative complications, postoperative hospital stay, and hospitalization expenses (25, 26). In patients with cholangitis, drainage of biliary obstruction by ERCP followed by laparoscopic cholecystectomy is the treatment of choice (11).

The overall findings from our study are consistent with those reported in other studies (11, 12), supporting the fact that LCBDE is associated with decreased postoperative discomfort, decreased postoperative hospital stay, and less morbidity, not only in young patients but also in the elderly. However, a larger prospective randomized controlled trial in the future would further help in assessing the long-term complications of LCBDE as well as in comparing the transcystic and transcholedochal approaches, which were beyond the scope of the present study. Other limitations of our study include the small sample size, lack of lithotripsy facilities, and the single-institution design. However, within our academic center, there has been a gradual shift toward laparoscopic management over ERCP for common bile duct (CBD) stones as part of our residency training program, including in patients with multiple stones. To the best of our knowledge, this represents the first study of its kind conducted in our country.

## 5 Conclusion

The present study establishes transcholedochal laparoscopic common bile duct exploration as a safe, effective, and versatile procedure for the management of CBD stones. It demonstrates excellent outcomes in terms of stone clearance, perioperative safety, low rates of major and minor complications, minimal need for open conversion, and low recurrence during follow-up. Notably, the procedure is feasible even in complex cases, including patients with multiple CBD stones (up to 10 stones in a single patient), markedly dilated ducts (up to 15 mm in diameter), and large individual stones (up to 16 mm in size). These findings emphasize that transcholedochal laparoscopic exploration can be confidently adopted as the treatment of choice for CBD stones, extending its benefits to a wider range of patients and reducing reliance on more invasive approaches.

## Data Availability

The original contributions presented in the study are included in the article/Supplementary Material, further inquiries can be directed to the corresponding author.
